# Effect of electroacupuncture on postoperative cognitive dysfunction for patients undergoing total knee arthroplasty: A protocol for systematic review and meta-analysis of randomized controlled trials

**DOI:** 10.1097/MD.0000000000023891

**Published:** 2021-01-29

**Authors:** Zixuan Ye, Mingjing Ke, Tao Wang, Yingxin Guan, Liang Ou, Linbiao Zheng, Zehua Chen, Zhen Shen, Liuyi Zheng, Changfei Yuan, Wenyao Li, Jinqing Liu, Yuheng Li, Shaodan Zhang, Huai Wu, Wengang Liu, Xuemeng Xu

**Affiliations:** a The Fifth Clinical Medical School, Guangzhou University of Chinese Medicine; b Kunming Municipal Hospital of Traditional Chinese Medicine, The Third Affiliated Hospital of Yunnan University of Chinese Medicine, Kunming; c Foshan Hospital of Traditional Chinese Medicine, Foshan; d The Second Affiliated Hospital of Guizhou University of Chinese Medicine, Guiyang; e Guangdong Second Traditional Chinese Medicine Hospital, Guangzhou, China.

**Keywords:** electroacupuncture, postoperative cognitive dysfunction, protocol, systematic review, total knee arthroplasty

## Abstract

**Background:**

Electroacupuncture is increasingly used in rehabilitation for postoperative cognitive dysfunction (POCD), but relevant evidence remains unclear for patients receiving total knee arthroplasty (TKA).

**Methods:**

The databases research of PubMed, EMBASE, CINAHL, and China National Knowledge Infrastructure (CNKI) will be conducted from inception to December 31, 2020. The relevant randomized controlled trials (RCTs) from data will be screened one by one. The remaining studies that meet the inclusion criteria will be extracted and analyzed using RevMan V.5.3 software. Paired 2 reviewers will assess quality of the included studies and publication bias by using the Cochrane Collaboration risk of bias tool, and Egger test and Begg test respectively. And grading of recommendations assessment, development and evaluation (GRADE) will be used to estimate the quality of evidence.

**Results:**

In this study, we will analyze the effect of electroacupuncture on Mini-Mental State Examination (MMSE), interleukin 1β (IL-1β), tumor necrosis factor-α (TNF-α), S100-β protein, and adverse events for patients with TKA.

**Conclusion:**

Our findings will provide evidence for the effectiveness of electroacupuncture on the treatment and prevention of POCD for TKA patients.

**Registration number:**

Available at: https://osf.io/azyt9 (DOI number: 10.17605/OSF.IO/AZYT9).

## 1 Introduction

Total knee arthroplasty (TKA) is considered as an effective treatment of end-stage knee osteoarthritis (KOA) due to the high recovery quality of function, significant pain relief, which makes KOA patients live a better life, and hence results in a high degree of patient satisfaction.^[1]^ Nevertheless, the complications after TKA still have focused increasing attentions, including postoperative pain, infection, prosthesis loosening, and anesthesia-related side effects like postoperative nausea, vomiting, cognitive dysfunction.^[2]^ Postoperative cognitive dysfunction (POCD), as one of the most common anesthesia-related complications, characterized by appears deficits in attention, memory, and comprehension, and even personality changes,^[3]^ which will decrease patient's postoperative satisfactory, and even result in delayed recovery. According to the data published, POCD shows a high prevalence ranges from 25% to 40% among elderly patients after surgery.^[4]^ Moreover, people suffering from POCD are often disoriented because of the decreased awareness of outside environment,^[5]^ which will put them in danger. Most of the patients receiving TKA are the elderly population which is at a high risk of POCD.^[6]^ Thus, it should not be neglected due to the series of unpleasant effects after TKA.^[7]^ Recently, there are many clinical trials^[8–10]^ conducted to seek an effective approach to reduce the incidence of POCD after TKA.

Electroacupuncture is applied to promote postoperative rehabilitation after TKA worldwide recently. As was previously reported, electroacupuncture presented positive effects on pain reduction,^[11]^ decreased ratio of nausea and vomiting during the postoperative recovery period.^[12]^ Furthermore, it was suggested that, compared with the basic therapies, the percentage of POCD presented a lower trend when the TKA patients treated with combination of electroacupuncture and basic therapies, while the assessment score for POCD was significantly lower.^[13]^ However, even though increasing number of studies aimed to investigate the effect of electroacupuncture on POCD after TKA, there is no evidence-based basis so far. Therefore, it is necessary to carry out a meta-analysis of randomized controlled trial (RCT) to examine the effectiveness of electroacupuncture on the POCD for the patients receiving TKA.

## 2 Methods

This meta-analysis will be performed according to the Preferred Reporting Items for Systematic Review and Meta-analyses.^[14]^ The protocol of this review has been registered in the Open Science Framework (OSF, https://osf.io/azyt9), and the registration DOI of this study is 10.17605/OSF.IO/AZYT9. We will submit any amendments to the present registered protocol along with the reasons for such changes to the database.

### 2.1 Selection criteria

#### 2.1.1 Study design

All the relevant articles of clinical RCTs examining the efficacy of electroacupuncture on POCD after TKA will be collected. We will include the clinical RCTs published in Chinese or English in a peer-reviewed journal. However, the articles including studies without full-text, unpublished literatures, observational studies, case series, case-control studies, basic experiments, qualitative studies, conferences, comments, and reviews will be excluded.

#### 2.1.2 Patients

In the present review and meta-analysis, the studies conducted in adult patients undergoing primary TKA will be considered. The patients receiving unicompartment knee arthroplasty or revision TKA will be excluded.

#### 2.1.3 Intervention

In order to understand the effect of electroacupuncture, 4 comparisons with respect to the interventions studied between experimental group and control group will be included in the present study: electroacupuncture versus basic treatment; electroacupuncture with basic treatment versus basic treatment; electroacupuncture versus other treatment; electroacupuncture versus placebo or sham electroacupuncture.

#### 2.1.4 Outcome measures

The ratio of POCD and Mini-Mental State Examination (MMSE) will be viewed as the primary outcomes of this review. Meanwhile, biochemical indicators related to changes of systematic inflammation status including interleukin 1β (IL-1β), tumor necrosis factor-α (TNF-α), S100-β protein, and adverse events will be included as secondary outcomes.

### 2.2 Search strategy

Database searches of PubMed, EMBASE, Web of Science, CINAHL, and China National Knowledge Infrastructure (CNKI) database will be performed by 2 searchers to identify the potentially relevant articles published from inception to December 31, 2020. A keyword such as “electroacupuncture,” “POCD,” “randomized controlled trial,” “randomized,” etc. will be used to search without restrictions. The key words which will be used in this study are listed in Table 1.

**Table 1:** Search strategies for CINAHL database.

No.	Query
#1	MeSH descriptor: [Postoperative Cognitive Complications] explode all trees
#2	(((((((((((((((((Postoperative Cognitive Dysfunctions)) OR (Cognitive Complication, Postoperative)) OR (Cognitive Complications, Postoperative)) OR (Complication, Postoperative Cognitive)) OR (Complications, Postoperative Cognitive)) OR (Postoperative Cognitive Complication)) OR (Postoperative Cognitive Dysfunction)) OR (Cognitive Dysfunction, Postoperative)) OR (Cognitive Dysfunctions, Postoperative)) OR (Dysfunction, Postoperative Cognitive)) OR (Dysfunctions, Postoperative Cognitive)) OR (Postoperative Cognitive Dysfunctions)) OR (Postoperative Decline)) OR (Decline, Postoperative)) OR (Declines, Postoperative)) OR (Postoperative Declines)):ti,ab,kw (Word variations have been searched)
#3	MeSH descriptor: [Electroacupuncture] explode all trees
#4	(Electroacupuncture):ti,ab,kw (Word variations have been searched)
#5	#1 OR #2
#6	#3 OR #4
#7	#5 AND #6

### 2.3 Study selection and data extraction

**Figure 1:**
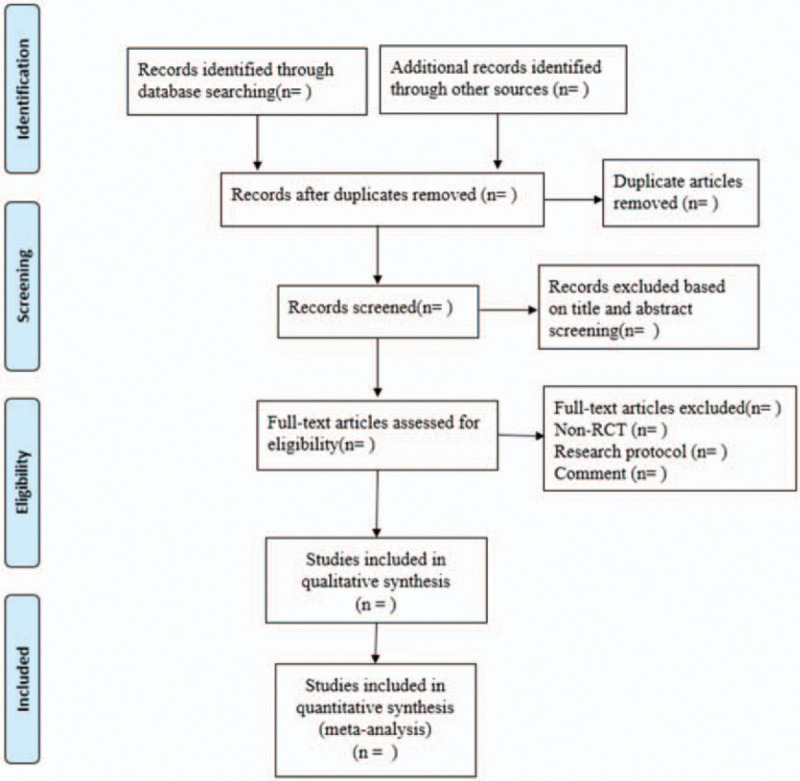
Flow chart of the search process.

In this study, paired investigators will screen all the literatures independently (LBZ and ZHC). Firstly, literatures will be preliminarily selected after the title and abstract screening. Secondly, full text of the eligible articles will be assessed strictly against the inclusion and exclusion criteria to identify the included studies. Subsequently, the main information of the articles will be collected, including authors’ names, publication year, countries, age, and sex of patients, study design, intervention type, acupoints, intensity, frequency, intervention dose, main outcomes, and sample size. Any discrepancies will be resolved through discussion, or consultation with the primary reviewer (XMX) until final consensus achieved during the period of screening and data extraction. A preferred reporting items for systematic reviews and meta-analyses flow chart will be drawn to illustrate the study selection procedure (Fig. 1).

### 2.4 Quality assessment

Two independent reviewers will assess the quality of the included RCTs according to the risk of bias table.^[15]^ The literature will be evaluated from 7 aspects: sequence generation, allocation concealment, blind of participants and personnel, blind of outcome, incomplete outcome data, selective reporting, and other biases. The risk of bias is divided into 3 levels: high, unclear, and low.

### 2.5 Data synthesis and analysis

After qualitative syntheses, meta-analysis of the included studies will be undertaken. And qualitative data will be pooled together and analyzed by using the Review Manger 5.3 software (Copenhagen, Denmark), and the results will be illustrated by the forest map intuitively. The continuous variables will be pooled by standard mean differences (SMDs) or mean differences (MDs) with 95% confidence intervals (95% CI), whereas the categorical data will be expressed as odds ratios (OR) associated with 95% CI. The Cochran Q-test and *I*^2^ index^[16]^ will be employed to assess heterogeneity. An *I*^2^ statistic >50% is considered to be substantially heterogeneous, and a random effects models will be used; otherwise, a fixed effects models will be utilized for the meta-analysis. Subgroup analysis will be conducted by study design and anesthesia type. The difference is viewed as statistically significant when *P*-values are <.05.

### 2.6 Assessment of reporting biases

Publication bias will be assessed by the Begg and Egger tests.^[17]^ A *P*-value <.05 in Egger test or Begg test is considered statistically significant.

### 2.7 Confidence in cumulative evidence

In addition, the GRADE (grading of recommendations, assessment, development, and evaluation; version:3.6) approach will be used to assess the quality of evidence.^[18]^ The quality of each evidence will be graded as high, moderate, low, or very low. Disagreements will be resolved by consensus.

## 3 Discussion

POCD is a decline cognitive status, and occurs after major surgery with a long term, especially in elderly patients. As it reported, POCD considered to be closely associated with lots of risk factors, such as age, anesthesia type, postoperative opioid consumption, and so on.^[19]^ TKA has mainly been used for the patients with advanced KOA. It was reported that the prevalence of TKA was 2.92% at 60 years, 7.29% at 70 years, 10.38% at 80 years, and 8.48% at 90 years in the United States,^[20]^ which demonstrated a high risk of POCD among the patients receiving TKA. However, although there are continuously increasing explorations for the treatment of POCD, it is still challenging to find the most suitable treatment for it. Until now, no effective prevention strategies have been recommended explicitly.

In previously studies, it was suggested that electroacupuncture had a positive effect on improving POCD for the TKA patients.^[13]^ However, whether it is effective and safe to be applied to treat and prevent POCD is still lack of evidence. This study will be the first time to systematically review and quantify the efficacy and safety of electroacupuncture for POCD after TKA. We hope this study will provide reference for the treatment and prevention of POCD in the fields of nonoperative therapies for the patients undergoing TKA.

## Author contributions

**Conceptualization:** Zixuan Ye, Zehua Chen, Yingxin Guan, Wengang Liu.

**Data curation:** Huai Wu, Tao Jang, Mingjing Ke.

**Formal analysis:** Tao Wang, Zhen Shen.

**Funding acquisition:** Huai Wu, Wengang Liu, Xuemeng Xu.

**Investigation:** Changfei Yuan, Liuyi Zheng, Zixuan Ye.

**Methodology:** Wenyao Li, Jinqing Liu, Liaobiao Zheng.

**Review sponsor:** Xuemeng Xu, Wengang Liu.

**Software:** Zehua Chen, Yuheng Li, Zixuan Ye.

**Writing – original draft:** Zehua Chen, Zixuan Ye.

**Writing – review & editing:** Shaodan Zhang, Zhen Shen.

## Abbreviations

Abbreviations: GRADE = Grading of Recommendations Assessment, Development and Evaluation; POCD = postoperative cognitive dysfunction; RCT = randomized controlled trial; TKA = total knee arthroplasty.
